# Phase II Study of Paclitaxel in Patients With Soft Tissue Sarcomas

**DOI:** 10.1080/13577149778362

**Published:** 1997-06

**Authors:** Shreyaskumar R. Patel, Kaye A. Linke, M. Andrew Burgess, Nicholas E. Papadopoulos, Carl Plager, Jan Jenkins, Robert Benjamin

**Affiliations:** Department of Melanoma/Sarcoma Medical Oncology MD Anderson Cancer Center University of Texas PO Box 77, 1515 Holcombe Blvd Houston TX 77030 USA

## Abstract

*Purpose*. Patients with soft tissue sarcoma (STS) who have previously received standard chemotherapy including adriamycin (doxorubicin), ifosfamide, cyclophosphamide and DTIC (dacarbazine) have very limited therapeutic options. It is important to identify new drugs with some activity in this disease and we therefore undertook this trial to determine the antitumor activity of paclitaxel (Taxol).

*Methods*. We conducted a phase II study of paclitaxel in patients with STS who had received prior standard chemotherapy. Paclitaxel was administered at a starting dose of 200 mg m^-2^ as a 24-h infusion with STS premedication, every 21 days or upon hematologic recovery (absolute granulocyte count (AGC) ≥ 1500/*μ*l, platelets ≥ 100 000/*μ*l). Neupogen was not used routinely. The study was conducted based on a two-stage design proposed by Simon. Responses were assessed radiographically using standard criteria.

*Results*. Nineteen eligible patients were treated in the first stage of the study. The median age was 50 years (range 20–68 years), and there were nine females and 10 males with Zubrod performance status of 1 or 2. One patient achieved a minor response. Median AGC nadir was 0.1/*μ*l on day 12 with absolute neutropenia lasting 5 days. Median platelet nadir was 171 000/*μ*l on day 9. There were no grade 3/4 non-hematologic toxicities and no deaths related to treatment.

*Discussion*. Paclitaxel, at this dose and schedule, is well tolerated but inactive in this patient population.


*Sarcoma* (1997) 1, 95–97

ORIGINAL ARTICLE

### Phase II study of paclitaxel in patients with soft tissue sarcomas

SHREYASKUMAR R. PATEL, KAYE A. LINKE, M. ANDREW BURGESS, NICHOLAS E. PAPADOPOULOS, CARL PLAGER, JAN JENKINS & ROBERT BENJAMIN

*Department of Melanoma/Sarcoma Medical Oncology, MD Anderson Cancer Center, University of Texas, USA*

**Abstract**

*Purpose.* Patients with soft tissue sarcoma (STS) who have previously received standard chemotherapy including adriamycin (doxorubicin), ifosfamide, cyclophosphamide and DTIC (dacarbazine) have very limited therapeutic options. It is important to identify new drugs with some activity in this disease and we therefore undertook this trial to determine the antitumor activity of paclitaxel (Taxol).

*Methods.* We conducted a phase II study of paclitaxel in patients with STS who had received prior standard chemotherapy. Paclitaxel was administered at a starting dose of 200 mg $m^{-2}$ as a 24-h infusion with STS premedication, every 21 days or upon hematologic recovery (absolute granulocyte count (AGC) $\geq$ 1500/$\mu$l, platelets $\geq$ 100 000/$\mu$l). Neupogen was not used routinely. The study was conducted based on a two-stage design proposed by Simon. Responses were assessed radiographically using standard criteria.

*Results.* Nineteen eligible patients were treated in the first stage of the study. The median age was 50 years (range 20–68 years), and there were nine females and 10 males with Zubrod performance status of 1 or 2. One patient achieved a minor response. Median AGC nadir was 0.1/$\mu$l on day 12 with absolute neutropenia lasting 5 days. Median platelet nadir was 171 000/$\mu$l on day 9. There were no grade 3/4 non-hematologic toxicities and no deaths related to treatment.

*Discussion.* Paclitaxel, at this dose and schedule, is well tolerated but inactive in this patient population.

**Key words:** *soft tissue sarcomas, chemotherapy, paclitaxel (Taxol).*

### Introduction

Paclitaxel (Taxol; Bristol-Meyers Squibb Co., Princeton, NJ, USA) is a novel antimicrotubule agent with a unique mechanism of action. It has been studied in a wide variety of malignancies with varying levels of efficacy.[1] Given its 'broad spectrum' activity, we performed a phase II study of paclitaxel in patients with soft tissue sarcoma (STS) who had received prior standard chemotherapy. Since leiomyosarcomas of gastrointestinal (GI) origin do not respond well to standard chemotherapy including adriamycin and ifosfamide, patients with this histology were eligible without prior exposure to chemotherapy. The major objectives of the study were to evaluate the efficacy and the toxicity profile of paclitaxel in patients with STS.

### Subjects and methods

### *Eligibility*

Patients older than 16 years of age with histologic proof of a STS were eligible. All patients had received prior standard chemotherapy including adriamycin, ifosfamide, cyclophosphamide and DTIC (dacarbazine) with the exception of patients with GI leiomyosarcoma who could be chemotherapy naïve. Patients were required to have a Zubrod performance status of 0–2, a life expectancy of at least 12 weeks, measurable or evaluable disease, and relatively normal organ function defined as absolute granulocyte count (AGC) of $\geq$ 1500/$\mu$l, platelet count of $\geq$ 100 000/$\mu$l, total bilirubin $<$ 1.5 mg $dl^{-1}$ serum creatinine $<$ 1.6 mg $dl^{-1}$ and cardiac ejection fraction of $>$ 50% without evidence of congestive heart failure. No other concurrent therapy was allowed and all patients signed an informed consent. Exclusion criteria included pregnancy, serious non-malignant intercurrent illnesses, serious conduction abnormalities or cardiac arrhythmias requiring anti-arrhythmic medications.

### *Treatment plan*

To minimize the risk of anaphylactoid reactions, all patients were premedicated with 20 mg of dexamethasone given orally, 12 and 6 h prior to paclitaxel, and 50 mg of diphenhydramine and 300 mg of

Correspondence to: S. R. Patel, University of Texas, MD Anderson Cancer Center, PO Box 77, 1515 Holcombe Blvd, Houston, TX 77030, USA. Tel: + 1 713 794 4632; Fax: + 1 713 794 1934; E-mail: spatel@notes.mdacc.tmc.edu.

1357-714X/97/020095-03 $9.00 ©1997 Carfax Publishing Ltd

**Table 1.** *Dose modification scheme*

| Dose levels ($mg\ m^{-2}$) | | Criteria for hematologic toxicity: Granulocyte nadir/µl | Platelet nadir/µl | Modification | Criteria for non-hematologic toxicity: Grade | Dose modification |
|---|---|---|---|---|---|---|
| −2 | 150 | >2000 | >100 000 | Increase 2 levels | 0–1 | Increase 1 level |
| −1 | 175 | 1000–2000 | 75 000–100 000 | Increase 1 level | 2 | No change |
| 0 | 200 | <500* | <50 000** | Decrease 1 level | 3 | Decrease 1 level |
| +1 | 225 | | | | 4 | Decrease 2 levels or stop |
| +2 | 250 | | | | | |

*Significant morbidity: organ infection, sepsis syndrome, failure to recover to ≥1500/µl by day 28.
**Significant morbidity: bleeding, platelet transfusions for ≥7 days, failure to recover to ≥100 000/µl by day 28.

cimetidine were given intravenously 1 h prior to paclitaxel. The starting dose of paclitaxel was 200 mg $m^{-2}$ administered as a 24-h continuous intravenous infusion via a central venous catheter. Cycles were repeated every 21 days or upon complete recovery. Granulocyte colony-stimulating factor was not used prohylactically, but was allowed for therapeutic indications. Dose modification was performed based on hematologic and non-hematologic toxicities as outlined in Table 1.

*Pretreatment evaluation and follow-up studies*

Prior to enrolment on the protocol, all patients underwent a complete history and physical examination. Laboratory studies included a complete blood count (CBC) with differential and platelets, chemistry profile (SMA 12), electrolytes and magnesium, repeated prior to each cycle. Appropriate radiographic studies were performed to define the extent of tumor. A cardiac scan or 2D echocardiogram was performed to document the cardiac ejection fraction prior to initiation of treatment. Following paclitaxel, patients were followed with at least once weekly CBC with differential and platelet counts. Cross-sectional radiographic imaging to assess response was performed every two cycles.

*Response criteria*

Complete response (CR) was defined as the disappearance of all clinical evidence of tumor. Partial response (PR) was defined as a ≥50% reduction in the sum of the products of the biperpendicular diameters of measurable lesions without the appearance of new lesions for at least 3 weeks. Minor response (MR) was defined as a decrease in tumor size between 25 and 49%. Stable disease (SD) was defined as a <25% change in the dimensions of the tumor and progressive disease (PD) was defined as a ≤25% increase in the sum of the perpendicular diameters and/or appearance of new lesions. Tumors that could not be bidimensionally measured were deemed evaluable. In these instance, regression, progression or no change were defined as unequivocal decrease, increase or no change in the tumor size and volume as agreed upon by two independent investigators. Development of new disease was also considered progression of disease.

*Statistical considerations*

The trial was conducted in two stages, using the optimal two-stage design proposed by Simon.[2] Based on the hypothesis that a response rate of ≤5% would be of no interest and a response rate of ≥20% would be significant, 15 patients were required and 19 patients were entered in the first stage of the study. If no responses were seen in these patients, the study would have to be terminated, otherwise a total of 35 patients needed to be accrued. This design afforded a power of 92% to detect a response rate of at least 20% with a rejection error of 10%.

### Results

*Patient characteristics*

Nineteen patients were entered on the study (Table 2). The median age was 50 years (range 20–68 years). There were nine females and 10 males with a Zubrod performance status of 1 or 2. Eight patients had leiomyosarcoma, three had unclassified sarcoma, three had stromal sarcoma of the breast, two had pleomorphic rhabdomyosarcoma, and one each had alveolar soft-part sarcoma, malignant fibrous histiocytoma and synovial sarcoma. Seventeen patients had metastatic disease and two had persistent or locally recurrent disease. Median number of cycles of paclitaxel chemotherapy administered was two (range 1–7).

*Response*

No objective CR or PR was seen. One patient with metastatic lung disease from a high-grade stromal sarcoma of the breast had an MR. Seven other patients had SD while 11 patients had PD.

**Table 2.** *Patient characteristics*

| Characteristic | Number |
|---|---|
| Patients entered | 19 |
| Age, years | |
| Median | 50 |
| Range | 20–68 |
| Zubrod performance status | |
| 1 | 18 |
| 2 | 1 |
| Sex | |
| Male | 10 |
| Female | 9 |
| Race | |
| Oriental | 2 |
| Other | 1 |
| White | 16 |
| Histology | |
| Leiomyosarcoma | 8 |
| Gastrointestinal | 6 |
| Uterus/retroperitoneal | 2 |
| Unclassified | 3 |
| Stromal sarcoma of breast | 3 |
| Others | 5 |
| Disease status | |
| Metastases | 17 |
| Uncontrolled primary or local recurrence | 2 |
| Prior therapy | |
| Surgery | 14 |
| Radiation | 3 |
| Chemotherapy | 12 |

*Toxicity*

Forty-one courses were administered at dose level 0 and two courses each were administered at dose levels 1 and −1. The National Cancer Institute's common toxicity criteria were used to assess toxicities. The median AGC nadir was 100/$\mu$l on day 12 of the cycle, lasting a median of 7 days (range 2–12) and the median platelet count nadir was 171 000/$\mu$l on day 9 of the cycle. Only five cycles were complicated by febrile neutropenia. There were no grade 3–4 non-hematologic toxicities and no deaths related to treatment.

### Discussion

According to the American Cancer Society's estimates, approximately 6400 new STS were diagnosed in 1996.[3] Chemotherapeutic agents with significant activity in these tumors include adriamycin, ifosfamide, cyclophosphamide and DTIC. Patients whose disease recurs or progresses after a trial of these standard agents have a guarded prognosis, and trials of new and interesting drugs are warranted. We have previously published our results with paclitaxel in bone sarcomas.[4] In this similar trial, we chose to evaluate the activity of pactitaxel at a dose of 200 mg $m^{-2}$ administered as a 24-h infusion, every 3 weeks in patients failing prior treatment with standard chemotherapy. No objective responses were noted in our trial suggesting a low level of activity of this drug in this patient population. A recently reported trial of paclitaxel in patients with previously untreated advanced STS revealed a response rate of 12.5%.[5] The patient population selected for our trial was refractory to standard chemotherapy, and therefore less likely to respond to paclitaxel. Six patients had leiomyosarcoma of GI origin which did not respond to paclitaxel.

In conclusion, paclitaxel at a dose of 200 mg $m^{-2}$ over 24-h every 3 weeks in patients with STS refractory to standard chemotherapy is well tolerated but inactive. Phase II trials of new agents are warranted.

### References

1 Rowinsky EK, Donehower RC. Drug therapy: paclitaxel (Taxol). *N Engl J Med* 1995; 332:1004–14.
2 Simon R. Optimal two-stage designs for phase II clinical trials. *Control Clin Trials* 1989; 10:1–10.
3 Parker SL, Tong T, Bolden S, *et al.* Cancer statistics 1996. *CA Cancer J Clin* 1996; 46:8.
4 Patel SR, Papadopoulos N, Plager C, *et al.* Phase II study of Paclitaxel in patients with previously treated osteosarcoma and its variants. *Cancer* 1996; 78:741–4.
5 Balcerzak SP, Benedetti J, Weiss GR, *et al.* A phase II trial of Paclitaxel in patients with advanced soft-tissue sarcomas—a Southwest Oncology Group Study. *Cancer* 1995; 76:2248–52.

## References

[PDF_00241] Balcerzak S. P., Benedetti J., Weiss G. R., Natale R. B. (1995). A phase II trial of paclitaxel in patients with advanced soft tissue sarcomas. A Southwest Oncology Group study.. Cancer.

[PDF_00238] Patel S. R., Papadopoulos N. E., Plager C., Linke K. A., Moseley S. H., Spirindonidis C. H., Benjamin R. (1996). Phase II study of paclitaxel in patients with previously treated osteosarcoma and its variants.. Cancer.

[PDF_00232] Rowinsky E. K., Donehower R. C. (1995). Paclitaxel (taxol). N Engl J Med.

[PDF_00234] Simon R. (1989). Optimal two-stage designs for phase II clinical trials.. Control Clin Trials.

